# Phenotyping spinal abnormalities in patients with Neurofibromatosis type 1 using whole-body MRI

**DOI:** 10.1038/s41598-021-96310-x

**Published:** 2021-08-19

**Authors:** Lennart Well, Anna Careddu, Maria Stark, Said Farschtschi, Peter Bannas, Gerhard Adam, Victor-Felix Mautner, Johannes Salamon

**Affiliations:** 1 Department of Diagnostic and Interventional Radiology and Nuclear Medicine, University Medical Center Hamburg-Eppendorf, Martinistraße 52, 20246 Hamburg, Germany; 2 Institute of Medical Biometry and Epidemiology, University Medical Center Hamburg-Eppendorf, Martinistraße 52, 20246 Hamburg, Germany; 3 Department of Neurology, University Medical Center Hamburg-Eppendorf, Martinistraße 52, 20246 Hamburg, Germany

**Keywords:** Musculoskeletal system, Neurological disorders, Epidemiology, Clinical genetics

## Abstract

Neurofibromatosis Type 1 (NF1) has been reported to be associated with a variety of spinal abnormalities. The purpose of this study was to quantify the prevalence of spinal abnormalities in a collective of NF1 patients that is representative for the general NF1 population, to associate the co-appearance of spinal abnormalities with both NF1 and clinical symptoms and to investigate if different mutations of the *NF1* gene affect the prevalence of these abnormalities. Retrospectively, 275 patients with NF1 and an age- and sex-matched collective of 262 patients were analyzed. The prevalence of spinal abnormalities was recorded. Mutational analysis of the *NF1* gene was obtained in 235 NF1 patients. Associations between spinal abnormalities, clinical symptoms and genotype were investigated by binary logistic regression analysis. Prevalence of all spinal abnormalities was higher in NF1 patients than in the control group. Six characteristics of spinal abnormalities were significantly associated with NF1 (all *p* < 0.05). An influence of scalloping on scoliosis (OR 3.01; *p* = 0.002); of meningoceles (OR 7.63) and neuroforaminal tumors (OR 2.96) on scalloping, and of dural ectasia on neuroforaminal tumors (OR 1.93) was identified. Backpain and loss of motor function were associated with neuroforaminal tumors, spinal tumors and scalloping of vertebral bodies (all *p* < 0.05). Specific mutations of the *NF1* gene were not relevantly associated with the development of spinal abnormalities. These findings can aid clinicians to improve clinical care of NF1 patients by creating awareness for co-appearences of specific spinal abnormalities and associated symptoms.

## Introduction

Neurofibromatosis type 1 (NF1) is an autosomal-dominantly inherited tumor predisposition syndrome with an incidence of 1 in 2500 newborns^1^. NF1 is caused by a mutation of the *NF1* gene on chromosome 17. The gene product of the *NF1* gene is Neurofibromin, which acts as a tumor suppressor and is an important factor for skeletal development^2^. Hallmark features of NF1 are growth of peripheral nerve sheath tumors, development of café au lait spots, axillary freckling, iris hamartomas and developmental skeletal abnormalities^3–7^.

Skeletal abnormalities have been mentioned in early reports on NF1 and include a variety of abnormalities, such as scoliosis, bowing of long bones, development of pseudarthroses and sphenoid wing dysplasia^5,8,9^. Especially spinal disorders have been identified as frequent skeletal abnormalities in NF1 patients, with scoliosis being the most common with a prevalence ranging from 10 to 60%^10–13^. Other previously described spinal abnormalities are scalloping of vertebral bodies, dural ectasia, meningoceles and widening of nerve root sleeves^14,15^.

The underlying cause for these osseous abnormalities in NF1 patients is not sufficiently understood^16^. Proposed reasons are a disorder of vitamin D homeostasis in NF1 patients^17^ and erosion of bones due to tumor growth^18–20^. Additionally, an increase in intradural pressure has been discussed as a cause for osseous abnormalities such as scalloping or dural ectasia whereas other authors suspect that osseous and especially vertebral abnormalities are a primary phenomenon of NF1 caused by mesodermal dysplasia^14,21,22^. Overall, the bone mineral density of NF1 patients is reduced^23,24^. Consecutively, the lifetime fracture frequency of these patients is increased^25^.

In recent years, correlations of genotype and phenotype in NF1 patients have been identified^26,27^. For example, a deletion of the whole *NF1* gene and its flanking regions, termed type-1 microdeletion, can result in a more severe course of the disease with an increase in development of cutaneous neurofibromas, frequent cognitive abnormalities, and a marfanoid habitus of patients, whereas a small three base-pair in-frame deletion in exon 17 leads to a mild manifestation of NF1 which is solely associated with pigmentary features^26,28^. Furthermore, missense and splice-site mutations have been associated with spinal neurofibromas^29^.

Most of the previous work on skeletal abnormalities in NF1 patients has been performed on small patient collectives (n < 30)^21^, without comparison against control groups^30^, or was focused on specific osseous abnormalities^31^. Larger patient collectives have been investigated by Jaremko et al. and Shah et al., however the spinal abnormalities quantified in these studies were solely scoliosis or dural ectasia and no correlation with other spinal abnormalities or clinical symptoms has been investigated^31,32^. Importantly, the patient collectives investigated in these previous studies do not necessarily represent the general population of NF1 patients. Patients included in these studies were recruited during treatment of spinal deformities^30^ or were enrolled in clinical trials^32^. An association between the phenotype of NF1 patients regarding spinal abnormalities and the underlying mutation of the *NF1* gene has been investigated^33^. However, the study of Waqar et al. was performed on a specific subset of NF1 patients with spinal neurofibromas which did not represent the general NF1 population and no other spinal abnormalities were included^33^.

Therefore, the purpose of this study was to quantify the prevalence of spinal abnormalities in a collective of NF1 patients that is representative of the general NF1 population, to associate the co-appearance of spinal abnormalities with both NF1 and clinical symptoms and to investigate if different mutations of the *NF1* gene effect the prevalence of these abnormalities.

## Methods

This retrospective, single-center, Health Insurance Portability and Accountability Act (HIPAA)-compliant study has been approved by the ethical-review board of the “Ärztekammer Hamburg” (No. PV7214) and complied with the local data protection guidelines as well as the Declaration of Helsinki. Written informed consent was obtained from all participants or their legal guardians.

### Patient population

Patients of the study group were selected from all patients that presented in our outpatient neurofibromatosis clinic between September 2014 and July 2017. Inclusion criteria were a diagnosis of NF1 according to the NIH criteria^34^ and availability of whole-body MRI examinations with axial, coronal and sagittal T2-weighted MRI sequences of the spine. The study group was compared to an age- and sex-matched control group without NF1. Inclusion criteria for the control group were: no known syndromal disease or connective tissue disorder (e.g. NF1 or 2, achondroplasia, mucopolysaccharidosis, Marfan syndrome, Ehlers-Danlos Syndrome) and availability of whole-spine MRI examinations with axial, coronal and sagittal T2-weighted sequences. Reasons for referral of the control group were suspected spondylodiscitis, multiple sclerosis or suspicion of spinal tumors. If available, mutations of the *NF1* gene were identified by sanger sequencing for patients of the study group, as described elsewhere^35,36^.

### Magnetic resonance imaging and image evaluation

Whole-body MR imaging was performed at 1.5 T (Siemens Magnetom, Siemens Healthineers, Erlangen, Germany) between September 2014 and July 2017. The following sequences were acquired: T1w TSE coronal (TR 731 ms; TE 11 ms; FA 160°; Matrix 512 × 448; FOV 500 × 400 mm; ST 7 mm; IG 8.75 mm), T2w TIRM coronal (TR 8850 ms; TE 44 ms; FA 180°; Matrix 384 × 384; FOV 499 × 399 mm; ST 3 mm; IG 3.6 mm), T2w TIRM axial (TR 4999 ms; TE 43 ms, FA 180°; Matrix 320 × 320; FOV 300 × 300 mm; ST 4 mm; IG 5.2 mm), T2w TIRM sagittal (TR 4880 ms; TE 51 ms; FA 180°; Matrix 512 × 512; FOV 400 × 400 mm; ST 3.0; IG 3.3), T1w TSE sagittal (TR 697 ms; TE 12 ms; FA 180°; Matrix 512 × 512; FOV 400 × 400 mm; ST 3.0; IG 3.3), and T2w TSE sagittal (TR 4600 ms; TE 96 ms; FA 160°; Matrix 512 × 504; FOV 350 × 320 mm; ST 3 mm) sequences. Intravenous contrast material was not administered.

### Image analysis

All images were read in consensus by two radiologists with 7 (LW) and 9 years (JS) of experience in MR imaging. Readers were blinded to whether a patient had NF1. Measurements were obtained on T2-weighted sequences.

### Scoliosis

**Figure 1:**
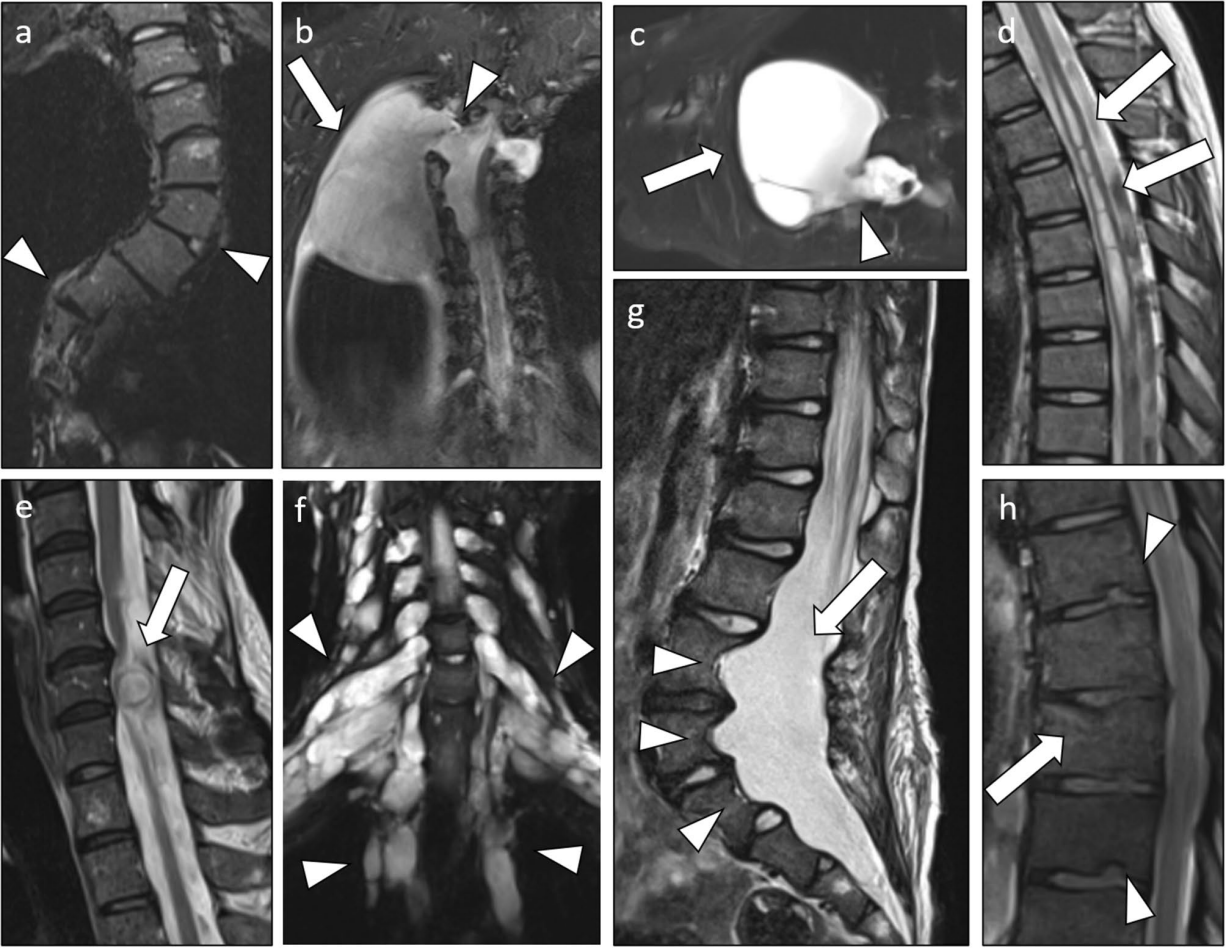
T2-weighted MRIs of investigated spinal abnormalities and tumors in patients with Neurofibromatosis type 1. Displayed are: A coronal image of a biconvex scoliosis of the thoracal spine (**a**). Coronal (**b**) and axial (**c**) images of a large meningocele (arrow) protruding through the enlarged neuroforamen of Th3 (arrowhead) into the thorax. A sagittal image of a septate thoracal syringomyelia (arrows) (**d**), a sagittal image of an intraspinal tumor at level C6 with compression of the spinal cord (**e**) and a coronal image of a patient with large paraspinal plexiform neurofibromas (arrowheads) growing along the whole spine (**f**). A sagittal image of a large dural ectasia (arrow) with scalloping of the lumbar vertebrae (arrowheads) (**g**) and a sagittal image of a vertebral fracture (arrow) and herniations of intervertebral discs into adjacent vertebrae (arrowheads) (**h**).

Extent of scoliosis was measured on coronal T2 weighted sequences (Fig. 1a). The extent of supine spinal lateral curvature was evaluated, based on curve direction (left/right convex or biconvex), curve location (cervical, thoracic, lumbar) and extent of curvature. For this purpose, Cobb angles between perpendiculars to endplates were measured at the upper and lower vertebrae forming the respective spinal curve^37^. Scoliosis was considered to be present in curves with Cobb angles greater than 10 degrees^32^.

### Meningoceles and syringomyelia

Lateral meningoceles were considered present when meningeal protrusions through neural foramina were detected (Fig. 1b, c)^38^. A syringomyelia was considered present, when cystic cavities within the spinal cord parenchyma were detected (Fig. 1d)^39^. Transversal and longitudinal diameters of both syringomyelias and meningoceles as well as the respective location in the spine were assessed.

### Dural ectasia and diameters of nerve root sleeves

For evaluation of dural ectasia, two different methods were applied. These have been proposed by Ahn et al. (dural ectasia_(Ahn)_) and Oosterhof et al. (dural ectasia_(Oost)_)^40,41^. The rationale for this approach was that prevalence of dural ectasia can vary considerably between these two methods which in turn can result in clinical treatment of patients^42^.

Ahn et al. defined two major and two minor criteria for the presence of dural ectasia^41^: Major criteria are width of the dural sac at a level below L5 greater than that above L4 or presence of an anterior sacral meningocele. Minor criteria are defined as scalloping greater than 3.5 mm at the level of S1 and nerve root sleeve diameters greater than 6.5 mm at the level of L5. Dural ectasia is considered to be present if one major or two minor criteria are found^41^. Therefore, sagittal diameters of the dural sac measured at levels L4 and S1 or below were compared and diameters of nerve root sleeves were evaluated on sagittal T2 weighted images. The maximum diameter of nerve root sleeves was measured for each foramen at the levels L1-S1. The mean for each level was calculated.

For evaluation of dural ectasia_(Oost)_, dural sac diameters and midsagittal diameters of vertebral bodies were obtained from L1-S1. The dural sac diameter was measured perpendicular to the long axis of the dural sac. The ratio of dural sac to vertebral body diameter was calculated for each level. Presence of dural ectasia was evaluated by using the following cut-off values proposed by Oosterhof et al. for levels L1-S1: 0.64, 0.55, 0.47, 0.48, 0.48 and 0.57^40^.

### Spinal tumors

The prevalence of intraspinal or paraspinal tumors was evaluated in all patients (Fig. 1e, f)^43^. Neurofibromas were identified by characteristic appearance as sharply delineated signal intense masses on T2-weighted sequences^44,45^. Tumor location and extent were measured.

### Scalloping

Scalloping was defined as a central erosion of the vertebral body as seen in the sagittal plane (Fig. 1g)^46^ and has been associated with dural ectasia in previous studies^41,47,48^. Scalloping was quantified by measuring the superior sagittal, midsagittal and inferior sagittal diameters of L1-S1^41,46^. The mean diameter from superior and inferior diameters was calculated and the midsagittal diameter was subtracted from this value. Measurements were obtained in the sagittal plane from the outer cortical margins of the vertebral bodies.

An additional classification for evaluation of dural ectasia and scalloping has been proposed by Fattori et al., who differentiate between three degrees of dural abnormalities^49^: Grade 1—mild dural ectasia—defined by bulging of the dural sac and lack of epidural fat at the level of the posterior wall of one vertebral body, by the presence of small radicular cysts, or by the presence of both. Grade 2—moderate dural ectasia—defined by bulging of the dural sac and lack of epidural fat at the level of the posterior wall of two or more vertebral bodies and presence of large radicular cysts. Grade 3—severe dural ectasia—defined by presence of an anterior sacral meningocele. All patients were evaluated according to these criteria.

### Fractures of vertebral bodies and intravertebral disk herniation

The presence of vertebral compression fractures was evaluated on sagittal T2-weighted sequences. Presence of a fracture was considered, if the height of a vertebral body was reduced by ≥ 20% in the anterior, middle or posterior dimension or if a general reduction in height of ≥ 20% was present, compared to the height of the cranial and caudal vertebrae^50,51^. Prevalence of intravertebral disk herniations was evaluated (Fig. 1h).

### Clinical assessment

All NF1 patients were evaluated for clinical symptoms that can be attributed to spinal abnormalities or spinal tumors by VFM and SF. Additionally, all symptomatic patients with unresolved clinical complaints underwent neurophysiological testing to exclude peripheral origin of symptoms. Findings of back pain were subdivided in pain of the cervical, thoracal, lumbar or sacral spine. When the pain was reported to be diffuse it was graded as unspecific/whole spine. Impairment or loss of motor function as well as sensory deficits were recorded.

### Genetic analysis

The 235 patients with available analysis of the underlying mutation of the *NF1* gene were divided into five different groups according to their type and extent of mutation of the *NF1* gene^52^: Group 1: large deletions of the *NF1* gene with a size of 1.4 Mb, encompassing the entire Neurofibromin gene and its flanking regions, known as type-1 deletion^26^; group 2: splice mutations; group 3: missense mutations; group 4: nonsense or frameshift mutations and group 5: patients without a detectable mutation of the *NF1* gene^53^. Patients that were not tested for mutations of the *NF1* gene were considered as group 6.

### Statistical analysis

Prevalence of spinal abnormalities was evaluated in the NF1 group and control group. Prevalence of clinical symptoms was evaluated in the NF1 group. The primary analysis represents the evaluation of associations of spinal abnormalities and NF1. They were analyzed by Chi-Squared Tests. If the calculation of an odds ratio was not possible due to complete separation, a binary logistic regression analysis with Firth’s correction for rare events was performed^54^. The results of the primary analysis were adjusted for multiple testing by Bonferroni correction. They were considered as significant if the adjusted p-value is smaller than the two-sided significance level of 5%. Further analyses were considered to be exploratory. They were performed by binary logistic regression models. In case of quasi-complete separation Firth’s correction was used. Resulting *p* values can only be interpreted in a descriptive manner because they were not adjusted for multiple testing. Diameters of nerve root sleeves for each segment from L1 to S1 and measurements of scalloping of vertebral bodies from L1 to S1 were compared between the NF1 group and the control group by Mann–Whitney-*U* test. The results were considered as significant if the *p* value is smaller than the two-sided significance level of 5%.

Additional secondary analyses aimed to assess the association of spinal abnormalities or clinical symptoms with the underlying mutation of the *NF1* gene. A binary logistic regression analysis was performed with clinical items as dependent variables and the items genetic group, age and sex as independent variables. To investigate if scoliosis, scalloping, dural ectasia, meningoceles and neuroforaminal tumors influence each other’s appearance, binary logistic regression models were calculated with one parameter as the dependent variable and the remaining parameters as the independent variables, respectively. Influence of spinal abnormalities on backpain or loss of motor function and sensory deficits was evaluated by binary logistic regression analysis. In case of quasi-complete or complete separation, binary logistic regression analysis with Firth’s correction was applied. Analyses were performed with R version 4.0.2.

## Results

### Patient population

Between September 2014 and July 2017, 2695 patients presented themselves in our outpatient clinic and were eligible for inclusion in this study. Due to missing whole-body MRI examinations, 2420 patients were excluded from this study. The resulting study group included 275 children, adolescents and adults (140 female; mean age 26.9 years; range 1–72 years; study group). Mutations of the *NF1* gene were identified by sanger sequencing in 235 of these NF1 patients. The control group consisted of 262 patients (136 females; mean age 27.3 years; range 1–72 years) without NF1. In the control group, the following spinal pathologies were identified in addition to the investigated spinal abnormalities: Disseminated T2 hyperintense lesions of the spinal cord in patients with known multiple sclerosis (44/262), disseminated osseous metastases (15/262), intraspinal disc protrusions (9/262), intervertebral osteochondrosis (8/262), spondylodiscitis (6/262), carcinomatous meningitis (5/262), traumatic intervertebral disc lesion (2/262), epidural empyema (1/262), reconversion of bone marrow (1/262), spinal ischemia (1/262), edema of the spinal cord of unknown origin (1/262). 132/262 patients did not show additional spinal pathologies.

### Spinal abnormalities in NF1 patients and control group

All investigated spinal abnormalities showed a higher prevalence in the NF1 group than in the control group (Table 1). The most common spinal abnormalities identified in NF1 patients were dural ectasia_(Oost)_ (NF1: 85.5% vs. control: 61.8%) and dural ectasia _(Ahn)_ (NF1: 44.7% vs. control 23.3%), scoliosis (NF1: 46.9% vs. control: 5%), and neuroforaminal tumors (NF1: 39.6% vs. control: 0%). The most common spinal deformity identified in the control group was dural ectasia_(Oost)_ (61.8%) and dural ectasia_(Ahn)_ (23.3%), similar to the NF1 group. The third most common spinal deformity in the control group was herniation of the intervertebral discs (NF1: 27.6% vs. control: 23.7%). Additional prevalences are given in Table 1.

**Table 1:** Spinal abnormalities in NF1 patients and the control group.

Item	NF1	Control	Odds ratio	99.5% CI	*p*
Scoliosis	46.9% (129/275)	5% (13/262)	16.9	7.1–40.3	0.00
Meningocele	5.5% (15/275)	0% (0/262)	31.2	0–0.5	0.17
Dural ectasia_(Ahn)_	44.7% (123/275)	23.3% (61/262)	2.7	1.6–4.5	0.00
Dural ectasia_(Oost)_	85.5% (235/275)	61.8% (162/262)	3.6	2–6.6	0.00
Neuroforaminal tumor	39.6% (109/275)	0% (0/262)	345.3	1.3–10.4	0.0004
Intraspinal tumor	12.7% (35/275)	3.8% (10/262)	3.7	1.8–10.8	0.0036
Scalloping_(Fat)_	18.5% (51/275)	5% (13/262)	4.3	0.8–11.9	0.00
Vertebral fracture	6.9% (19/275)	2.3% (6/262)	3.1	0.3–4.6	0.21
Syringomyelia	3.6% (10/275)	3.1% (8/262)	1.2	0.7–2.1	1.0
Herniation of intervertebral disc	27.6% (76/275)	23.7% (62/262)	1.2		1.0

Presented are prevalence and association of spinal abnormalities with NF1. Due to Bonferroni adjustment for multiple testing, adjusted p-values and 99.5% confidence intervals are reported.

Values in parentheses are total numbers.

*CI* confidence interval.

The most common location of meningoceles in NF1 patients was the thoracic spine with 8/14 (80%) patients affected, compared to cervical (1/14; 7.1%), lumbar (4/14; 28.6%) and sacral (1/14; 7.1%) meningoceles. Nerve root sleeves were significantly enlarged in NF1 patients compared to the control group with mean values of L1-S1 ranging from 9.0 to 11.1 mm in the NF1 group and from 6.4 to 8.0 mm in the control group (Table 2). Patients with neuroforaminal tumors displayed widened neuroforamina in all lumbar segments compared to patients without neuroforaminal tumors (all segments *p* < 0.0001). Measurement of scalloping revealed a greater difference between midsagittal diameters and superior / inferior sagittal diameters in vertebral bodies of NF1 patients (2.7–3.3 mm) compared with control patients (1.9–2.4 mm) (Table 3).

**Table 2:** Measurement of diameters of nerve root sleeves in NF1 patients and the control group.

Segment		Nerve root sleeves							
		NF1						Control	*p*
		G1	G2	G3	G4	G5	G6	–	
L1	Subgroups	8.9 ± 2.1	8.5 ± 1.1	9.4 ± 1.8	8.9 ± 1.4	9.0 ± 1.8	8.8 ± 1.2	6.4 ± 1.4	< 0.0001
	Mean	9.0 ± 1.6							
L2	Subgroups	9.5 ± 1.9	9.1 ± 1.4	9.5 ± 1.7	9.2 ± 1.6	9.2 ± 1.9	9.1 ± 1.4	6.9 ± 1.5	< 0.0001
	Mean	9.3 ± 1.6							
L3	Subgroups	9.3 ± 1.9	9.1 ± 1.4	9.3 ± 1.6	9.4 ± 2.2	9.3 ± 2.0	8.9 ± 1.2	6.9 ± 1.5	< 0.0001
	Mean	9.3 ± 1.9							
L4	Subgroups	9.5 ± 2.0	9.2 ± 1.5	9.5 ± 1.6	9.4 ± 1.8	9.3 ± 2.0	9.0 ± 1.6	7.0 ± 1.5	< 0.0001
	Mean	9.3 ± 1.8							
L5	Subgroups	9.5 ± 1.9	9.9 ± 2.1	10.8 ± 3.0	10.2 ± 2.0	9.8 ± 2.4	9.4 ± 2.0	7.1 ± 1.5	< 0.0001
	Mean	10.1 ± 2.3							
S1	Subgroups	10.6 ± 2.7	11.2 ± 3.0	11.9 ± 3.4	11.2 ± 3.3	10.6 ± 3.4	10.9 ± 2.8	8.0 ± 1.8	< 0.0001
	Mean	11.1 ± 3.2							

Values represent mean values ± standard deviation. Values are given in mm.

G1: genetic subgroup 1 (type-1 deletion); G2: genetic subgroup 2 (splice mutation); G3: genetic subgroup 3 (missense); G4: genetic subgroup 4 (nonsense/frameshift mutation); G5: genetic subgroup 5 (no proof of mutation); G6: genetic subgroup 6 (no analysis performed).

**Table 3:** Measurement of vertebral scalloping in NF1 patients and the control group.

Segment		Scalloping of vertebrae							
		NF1						Control	*p*
		G1	G2	G3	G4	G5	G6	–	
L1	Subgroups	2.7 ± 1.5	3.5 ± 3.0	2.8 ± 1.3	2.6 ± 1.4	2.9 ± 2.0	2.7 ± 1.6	2.3 ± 1.6	0.02
	Mean	2.8 ± 1.7							
L2	Subgroups	2.3 ± 1.1	2.9 ± 1.4	2.7 ± 1.2	2.9 ± 1.5	2.8 ± 1.4	2.7 ± 1.9	2.4 ± 1.5	0.016
	Mean	2.8 ± 1.5							
L3	Subgroups	2.1 ± 1.3	3.2 ± 1.7	2.6 ± 1.5	2.9 ± 1.8	2.5 ± 1.4	2.6 ± 1.4	2.2 ± 1.5	0.01
	Mean	2.7 ± 1.6							
L4	Subgroups	3.1 ± 2.3	3.1 ± 1.7	2.7 ± 1.7	2.8 ± 1.9	2.5 ± 1.4	2.9 ± 2.1	2.2 ± 1.4	0.001
	Mean	2.8 ± 1.8							
L5	Subgroups	2.1 ± 1.1	3.2 ± 1.6	3.4 ± 1.8	2.7 ± 1.5	2.5 ± 1.4	2.5 ± 1.4	1.9 ± 1.5	< 0.0001
	Mean	2.7 ± 1.5							
S1	Subgroups	3.1 ± 1.3	3.2 ± 1.4	3.2 ± 1.9	3.3 ± 1.5	2.8 ± 1.4	3.3 ± 1.6	2.4 ± 1.5	< 0.0001
	Mean	3.3 ± 2.5							

Values represent mean values ± standard deviation. Values are given in mm.

G1: genetic subgroup 1 (type-1 deletion); G2: genetic subgroup 2 (splice mutation); G3: genetic subgroup 3 (missense); G4: genetic subgroup 4 (nonsense/frameshift mutation); G5: genetic subgroup 5 (no proof of mutation); G6: genetic subgroup 6 (no analysis performed).

### Spinal abnormalities and association with NF1

Significant associations between six parameters of spinal abnormalities and NF1 were identified. Due to the application of Bonferroni adjustment for multiple testing, adjusted p-values and 99.5% confidence intervals are reported. These six items were: scoliosis (OR 16.9; 99.5% CI 7.1–40.3; *p* < 0.0001), dural ectasia_(Ahn)_(OR 2.7; 99.5% CI 1.6–4.5; *p* < 0.0001), dural ectasia_(Oost)_(OR 3.6; 99.5% CI 2–6.6), neuroforaminal tumors (OR 345.3; 99.5% CI 0–6.3; *p* = 0.0004), spinal tumors (3.7; OR 1.3–10.4; *p* = 0.0036), and scalloping_(Fat)_(OR 4.4; 99.5% CI 1.6–10.8; *p* < 0.0001). Other associations between NF1 and spinal abnormalities did not reach significance (*p* > 0.05) (Table 1). Odds ratios of other investigated spinal abnormalities in NF1 patients are given in Table 1.

### Spinal abnormalities and genetic subgroups

**Figure 2:**
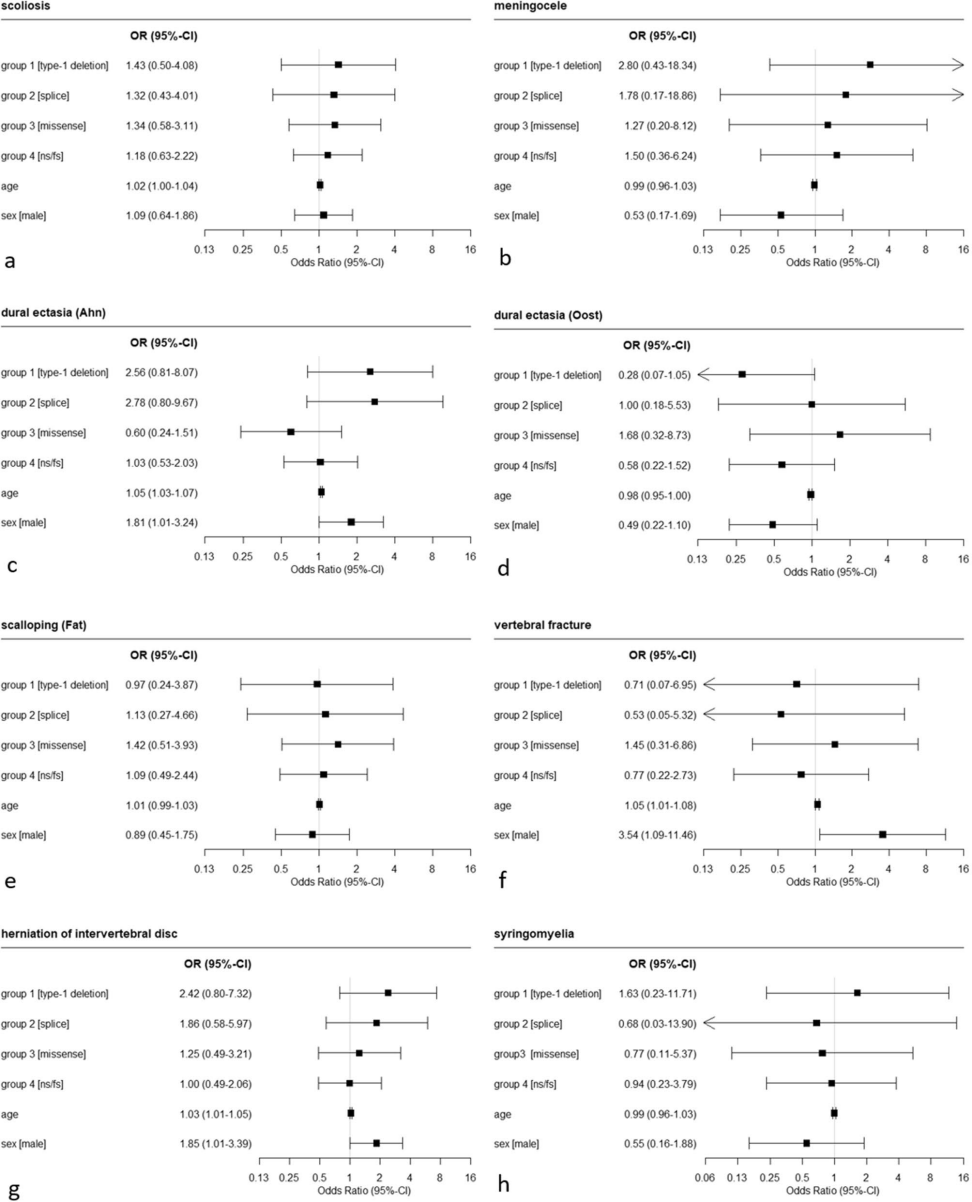
Results of logistic regression analysis on the association of spinal abnormalities with type of mutation of the *NF1* gene, age and sex.

**Figure 3:**
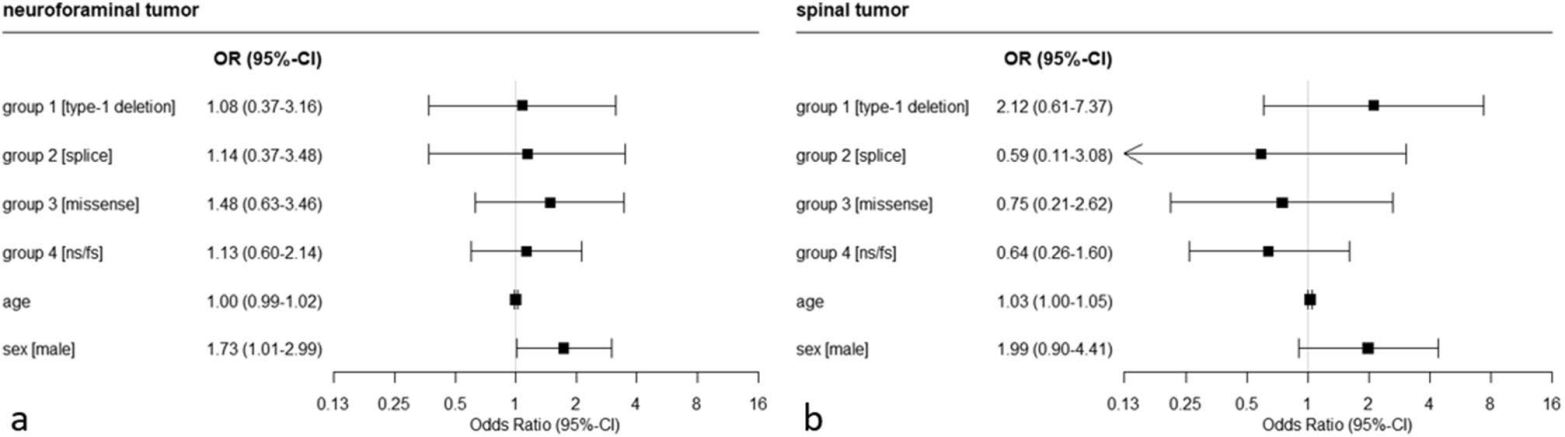
Results of logistic regression analysis on the association of neuroforaminal (**a**) and spinal (**b**) tumors with type of mutation of the *NF1* gene, age and sex.

Overall, the investigated spinal abnormalities displayed an almost evenly distribution between the genetic subgroups. No association between spinal abnormalities and a specific genetic subgroup was identified (Figs. 2, 3). The most common deformity in all genetic subgroups was dural ectasia_(Oost)_. Other abnormalities with high prevalence in the respective genetic subgroups were: group 1: dural ectasia_(Ahn)_ (61.1% ); group 2: dural ectasia_(Ahn)_ (68.8%); group 3: scoliosis (50%); group 4: scoliosis (50%); group 5: scoliosis and dural ectasia_(Ahn)_ (both 41.4%); group 6: scoliosis (52.5%). Associations of the genetic profile with investigated items did reveal large p-values of calculated odds ratios (Figs. 2, 3). More detailed information on the association of spinal abnormalities and genetic subgroups and prevalences of all spinal abnormalities within the different genetic subgroups are provided in Supplemental Table S1.

### Co-appearance of spinal abnormalities

The analysis to investigate if scoliosis, scalloping, dural ectasia, meningoceles and neuroforaminal tumors influence each other’s appearance showed relevant effects. Patients who suffered from scalloping of vertebral bodies had relevantly increased odds for development of scoliosis (OR 3.01; *p* = 0.002). Patients who suffered from scoliosis (*p* = 0.002; OR 3.13), meningoceles (OR 7.63; *p* = 0.001) or neuroforaminal tumors (OR 2.96; *p* = 0.002) had increased odds for development of scalloping of vertebral bodies. Suffering from scalloping of vertebral bodies relevantly increased odds for meningoceles (OR 7.7; *p* = 0.001) and for neuroforaminal tumors (OR 2.89; *p* = 0.002). The presence of dural ectasia_(Ahn)_ also increased odds to suffer from neuroforaminal tumors (OR 1.93; *p* = 0.011). Neuroforaminal tumors relevantly increased odds to suffer from dural ectasia_(Ahn)_ (OR 1.93; *p* = 0.011), whereas dural ectasia_(Oost)_ did not (OR 1.16; *p* = 0.69). More detailed results on co-appearance of these spinal abnormalities are provided in Supplemental Figure S1.

### Clinical symptoms in NF1 patients

The most common clinical symptom within the NF1 group was pain of the thoracic spine (29/275; 10.5%). Other common clinical symptoms were impairment or loss of motor function in 27/275 (9.8%) patients and pain of the lumbar spine (25/275; 9.1%). Further details on clinical symptoms overall and within the genetic subgroups are given in Table 4.

**Table 4:** Prevalence of clinical symptoms in NF1 patients and type of mutation of the *NF1* gene.

Clinical symptoms	NF1	Group 1 (type 1 del)	Group 2 (splice)	Group 3 (missense)	Group 4 (nonsense/frameshift)	Group 5 (no proof)	Group 6 (no analysis)
Cervical spine pain	4% (11/275)	0% (0/18)	0% (0/16)	0% (0/34)	5.2% (5/97)	4.3% (3/70)	7.5% (3/40)
Thoracic spine pain	10.5% (29/275)	11.1% (2/18)	6.3% (1/16)	5.9% (2/34)	12.4% (12/97)	12.9% (9/70)	7.5% (3/40)
Lumbar spine pain	9.8% (27/275)	5.6% (1/18)	12.5% (2/16)	2.9% (1/34)	11.3% (11/97)	10% (7/70)	12.5% (5/40)
Pelvic/sacral pain	1.5% (4/275)	0% (0/18)	12.5% (2/16)	0% (0/34)	1% (1/97)	0% (0/70)	2.5% (1/40)
Whole spine pain	2.9% (8/275)	5.6% (1/18)	6.3% (1/16)	5.9% (2/34)	1% (1/97)	4.3% (3/70)	0% (0/40)
Pain_(pooled)_	23.3% (64/275)	22.2% (4/18)	31.3% (5/16)	14.7% (5/34)	22.7% (22/97)	25.1% (18/70)	25% (10/40)
Loss of sensitivity	5.5% (15/275)	0% (0/18)	0% (0/16)	2.9% (1/34)	4.1% (4/97)	8.6% (6/70)	10% (4/40)
Loss of motor function	9.8% (27/275)	5.6% (1/18)	6.3% (1/16)	11.8% (4/34)	9.3% (9/97)	11.4% (8/70)	10% (4/40)

**Figure 4:**
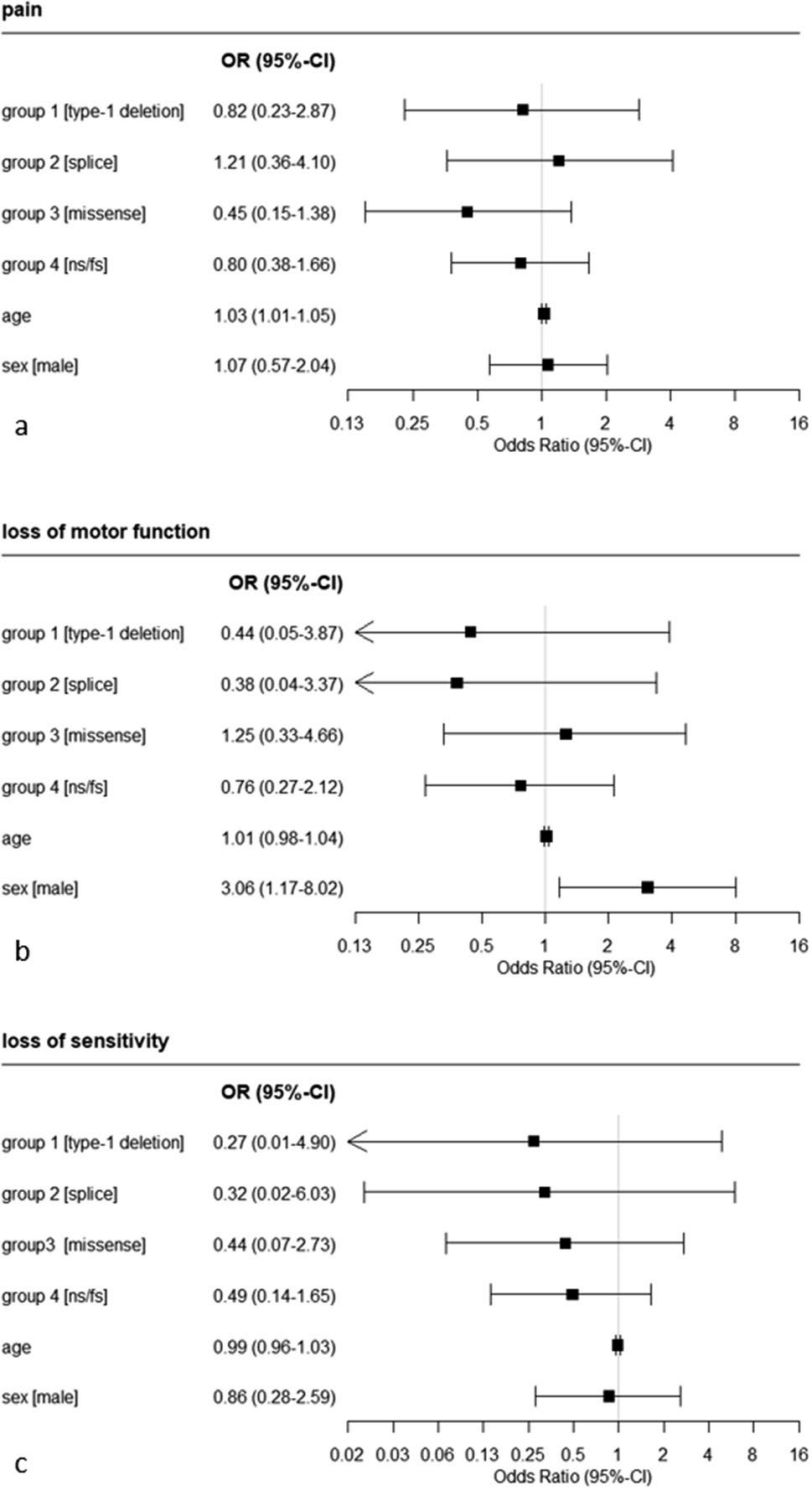
Results of logistic regression analysis on the association of clinical symptoms backpain (**a**), loss of motor function (**b**) and loss of sensitivity (**c**) with type of mutation of the *NF1* gene, age and sex.

Analysis for clinical symptoms and their association with NF1 showed that age influenced back pain (OR 1.03; *p* = 0.006) (Fig. 4 a) and that male sex influenced loss of motor function (OR 3.06; *p* = 0.023) (Fig. 4 b). None of the genetic subgroups displayed relevantly increased odds for the development of clinical symptoms (Fig. 4). More detailed results on clinical symptoms and their association with NF1 are provided in Supplemental Table S2.

### Influence of spinal abnormalities on clinical symptoms

**Figure 5:**
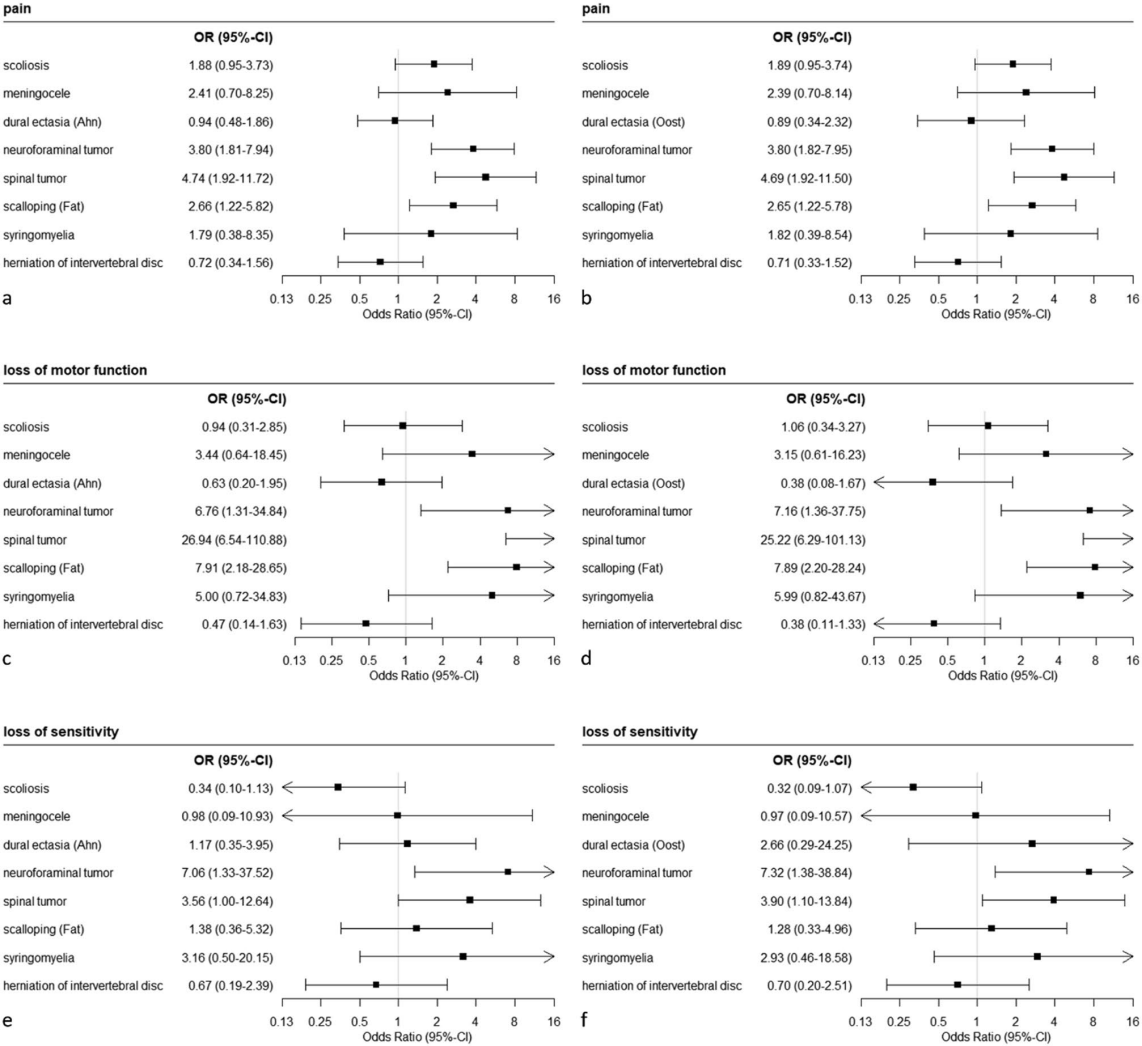
Results of logistic regression analysis on the association of spinal deformities with clinical symptoms. Results in the left column are based on measurements of dural ectasia as proposed by Ahn et al. Results in the right column are based on measurements as proposed by Oosterhof et al.

Logistic regression analysis revealed that spinal abnormalities exert influence on clinical symptoms. Neuroforaminal tumors (OR 3.8; *p* < 0.001), intraspinal tumors (OR 4.74; *p* = 0.001) and scalloping of vertebral bodies (OR 2.66; *p* = 0.014) relevantly increased the odds of back pain (Fig. 5a, b). Loss of motor function was also relevantly associated with neuroforaminal tumors (OR 6.76; *p* = 0.022), spinal tumors (OR 26.94; *p* < 0.001), and scalloping of vertebral bodies (OR 7.91; *p* = 0.002) (Fig. 5c, d). Loss of sensitivity was relevantly associated with presence of neuroforaminal tumors (OR 7.06; *p* = 0.022) or spinal tumors (OR 3.56; *p* = 0.05) (Fig. 5e, f). Prevalences of clinical symptoms and more details on the influence of spinal abnormalities on clinical symptoms are provided in Supplemental Table S3.

## Discussion

Prevalence of spinal abnormalities in NF1 patients was increased compared with the general population in this study. Development of scoliosis, neuroforaminal or spinal tumors and dural ectasia were significantly associated with NF1. Co-appearances of scoliosis, scalloping of vertebral bodies, meningoceles, neuroforaminal tumors, and dural ectasia were identified. No relevant association between mutations of the *NF1* gene and spinal abnormalities was revealed. Backpain and loss of motor function were associated with neuroforaminal tumors, spinal tumors and scalloping of vertebral bodies.

The comparison of spinal abnormalities in NF1 patients with the general population shows that NF1 patients display a higher prevalence of all investigated spinal abnormalities. Especially the prevalence of scoliosis (46.9% vs. 5%) and neuroforaminal tumors (39.6% vs. 0%) was markedly increased in the NF1 population when compared with the control group. In contrast, the prevalence of vertebral fractures (6.9% vs. 2.3%), syringomyelia (3.6% vs. 3.1%) and herniation of the intervertebral discs (27.6% vs. 23.7%) was only mildly increased in NF1 patients. The identified prevalence of scoliosis of 46.9% in this study is well within range with previous reports^6,10,12,13^ whereas the prevalence of neuroforaminal tumors (39.6%) was lower compared to the 79% identified by Nguyen et al.^43^. The only mildly increased prevalence of vertebral fractures and herniation of intervertebral discs does not seem to reflect previous reports on decreased bone density in NF1^24^. However, measurement of scalloping and nerve root sleeve diameters in our study showed increased abnormalities of vertebral bodies in NF1 patients in all lumbar segments. It remains unclear if these abnormalities are caused by increased intradural pressure or tumor growth or as a primary symptom of NF1. However, these findings are of importance in a clinical context, because scalloping has been associated with progression of scoliosis^55^.

The highest prevalence of spinal abnormalities within the group of NF1 patients was identified for dural ectasia as measured by the method proposed by Oosterhof et al. (85.5%)^47^. Prevalence of dural ectasia dropped when measured by the method proposed by Ahn et al. (44.7%)^41^. The method by Oosterhof et al. potentially overestimates the prevalence of dural ectasia as both, the NF1 group as well as the control group, displayed a very high prevalence of dural ectasia. This hypothesis has already been made by other authors and led to the development of a recently published grading system by Shah et al.^31^. With the method proposed by Shah et al. the prevalence of dural ectasia in NF1 patients investigated in that study was only 10%^31^. The correct assessment of dural ectasia is of importance because it has been associated with scalloping of vertebral bodies by other authors and if progressive, can lead to spinal instability requiring surgical stabilization^21,56,57^.

Prevalence of scoliosis was high in NF1 patients in this study (46.9%). The prevalence was higher than that reported by Jaremko et al. (20%) but was in line with other reports that ranged from 10 to 60%^10–13,15^ and has been described as the most common spinal deformity by other authors^10–13^. Due to potentially severe progression of scoliosis, patients have to be evaluated for surgical stabilization. Previous studies have demonstrated that patients with dystrophic features of the spine, e.g. vertebral scalloping, vertebral wedging, paravertebral soft tissue masses or neuroforaminal enlargement, have an increased risk for progression of scoliosis and should therefore be considered for surgery^10,12^. Furthermore, Durrani et al. found that presence of three or more dysplastic features significantly increases the risk for progression of scoliotic curvature^57^. An association of scoliosis with spinal abnormalities has been described. Nguyen et al. found that prevalence of scoliosis was associated with that of meningoceles^43^. In our patient collective, scoliosis was associated with scalloping of vertebral bodies. Evaluation of scoliosis is of importance due to the high prevalence and potentially significant effect on affected patients. Especially patients with additional dystrophic features of the spine should be thoroughly evaluated for surgical therapy.

The prevalence of neuroforaminal tumors in NF1 patients in this study (39.6%) was within the wide range of previously published studies (13.2–80%)^43,58,59^. As stated by Nguyen et al., the high prevalence of 80% in their study potentially is an overestimation regarding the general NF1 population and may be caused by the more severe phenotype of NF1 in the studied patient group^43^. Additionally, in the study by Nguyen et al. 60% of patients presented with clinical symptoms which is higher than that reported by NF1 patients in our study (back pain: 23.3%)^43^. Zhongshan et al. described an increased growth of spinal tumors along the concave side of scoliosis, an association that we were not able to confirm^60^. Neuroforaminal tumors have been discussed as a reason for widening of neuroforamina^43^. Our study supports this hypothesis due to the relevant widening of neuroforamina in patients with neuroforaminal tumors compared to patients without neuroforaminal tumors within the NF1 group (all segments *p* < 0.0001). Additionally, patients with neuroforaminal tumors and widened neuroforamina suffered from back pain more frequently than patients without neuroforaminal tumors. These findings further demonstrate the effect of spinal abnormalities on affected patients and emphasize the necessity to evaluate routinely performed MRI scans regarding these abnormalities.

Further associations between spinal abnormalities were identified in this study. We found associations between scoliosis, scalloping, meningoceles, neuroforaminal tumors and dural ectasia. Previous studies only showed associations between singular items, e.g. scoliosis and meningoceles^43^, an increased appearance of neuroforaminal tumors on the convex side of scoliosis or that patients with neurofibromas have increased odds for spinal curvature anomalies^30,43^. Shah et al. found that patients with dural ectasia have higher odds ratios for spinal abnormalities^31^. However, the method used to evaluate dural ectasia in that study was different from the methods used in our study. Most importantly, a recent study on the molecular basis of skeletal manifestations in NF1 found that bone surfaces adjacent to neurofibromas display increased areas of non-mineralization. Our data support this observation by revealing a relevant association of neuroforaminal tumors with scalloping of vertebral bodies and dural ectasia.

We identified an association of backpain and loss of motor function in NF1 patients with neuroforaminal tumors, intraspinal tumors and scalloping of vertebral bodies. These findings are of importance, as MRI can help to identify patients at risk of development of clinical symptoms. Therefore, routine spinal MRI examinations as part of standard clinical care in NF1 patients should be discussed. However, caution is required when evaluating the etiology of clinical symptoms in NF1 patients due to the multitude of manifestations of this disease.

The correlation of spinal abnormalities and clinical symptoms with specific mutations of the *NF1* gene did not yield small p-values. None of the investigated abnormalities was associated with a specific type of mutation and no mutation showed an increased prevalence of spinal abnormalities in affected patients. Similarly, a previous study on changes of the lung parenchyma in NF1 patients did not identify an association of specific gene mutations and pulmonary pathologies^52^. In contrast, other studies found that specifically patients with type-1 mutations of the *NF1* gene suffer from an increased whole-body tumor burden and that these patients develop large tumor volumes of > 3000 ml more frequently^61^. Furthermore, Ruggieri et al. found that missense mutations of the *NF1* gene are significantly associated with spinal neurofibromas^62^. Additionally, Cai et al. identified differently expressed genes in NF1 patients which may be involved in the development of spinal abnormalities^63^. These studies suggest that associations between genotype and phenotype indeed exist within the spectrum of NF1. However, due to the heterogeneous genetic background and various clinical manifestations of the disease, studies within a larger population of NF1 patients seem to be necessary to identify these correlations.

Our study has several limitations. First, the number of patients investigated with regard to specific mutations of the *NF1* gene was limited and larger studies might be needed to identify associations between genotype and spinal abnormalities. However, other studies investigating associations of genotype and phenotype in NF1 confirmed correlations in smaller cohorts^28,61,62^. Second, measurement of scoliosis was performed in a supine position. Measurement of scoliosis in supine MRI has been reported to underestimate Cobb angles^64^. Hence, the prevalence and severity of scoliosis might have been underestimated. However, Brink et al. demonstrated a significant association between conventional radiographs and supine MRI examinations for evaluation of idiopathic scoliosis^65^. Furthermore, the MRI examinations of NF1 patients in this study were wholy-body MRI scans, performed at 1.5 T. Diagnostic accuracy might be improved by use of dedicated spinal MRI examinations at 3 T with thinner slice thickness.

In conclusion, we were able to evaluate the prevalence of spinal abnormalities in a patient collective which is representative of the general NF1 population. Our results confirm that spinal abnormalities in NF1 patients have a higher prevalence compared to the general population. Development of scoliosis and neuroforaminal tumors are significantly associated with NF1 and co-appearances of scoliosis, scalloping of vertebral bodies, meningoceles, neuroforaminal tumors, and dural ectasia were identified. Backpain and loss of motor function were associated with neuroforaminal tumors, spinal tumors and scalloping of vertebral bodies. Specific mutations of the *NF1* gene were not relevantly associated with the development of spinal abnormalities. Therefore, the genetic profile of NF1 patients does not reliably predict prevalence and extent of spinal abnormalities. The findings of this study can aid clinicians to improve clinical care of NF1 patients by creating awareness for co-appearences of specific spinal abnormalities and associated symptoms.

## Supplementary Information

Supplementary Legends.

Supplementary Figure S1.

Supplementary Table S1.

Supplementary Table S2.

Supplementary Table S3.

## Data Availability

The datasets generated during and/or analysed during the current study are available from the corresponding author on reasonable request.
