# Author Correction: Phenotyping spinal abnormalities in patients with Neurofibromatosis type 1 using whole-body MRI

**DOI:** 10.1038/s41598-021-97577-w

**Published:** 2021-08-30

**Authors:** Lennart Well, Anna Careddu, Maria Stark, Said Farschtschi, Peter Bannas, Gerhard Adam, Victor-Felix Mautner, Johannes Salamon

**Affiliations:** 1 Department of Diagnostic and Interventional Radiology and Nuclear Medicine, University Medical Center Hamburg-Eppendorf, Martinistraße 52, 20246 Hamburg, Germany; 2 Institute of Medical Biometry and Epidemiology, University Medical Center Hamburg-Eppendorf, Martinistraße 52, 20246 Hamburg, Germany; 3 Department of Neurology, University Medical Center Hamburg-Eppendorf, Martinistraße 52, 20246 Hamburg, Germany

Correction to: *Scientific Reports* 10.1038/s41598-021-96310-x, published online 19 August 2021

The original version of this Article contained errors in the spelling of the authors Lennart Well, Anna Careddu, Maria Stark, Said Farschtschi, Peter Bannas, Gerhard Adam, Victor-Felix Mautner & Johannes Salamon which were incorrectly given as Well Lennart, Careddu Anna, Stark Maria, Farschtschi Said, Bannas Peter, Adam Gerhard, Mautner Victor-Felix & Salamon Johannes.

The original Article has been corrected.

