# Supplementary material for: Phenotyping spinal abnormalities in patients with Neurofibromatosis type 1 using whole-body MRI
Source: Sci Rep. 2021 Aug 19;11:16889. doi: 10.1038/s41598-021-96310-x (PMC8376946; doi:10.1038/s41598-021-96310-x)
Supplement: Supplementary file 1 — Supplementary Legends. [file 41598_2021_96310_MOESM1_ESM.docx]

**Supplemental figure legends:**

**Supplemental Figure S1:** Odds ratios of co-appearances of spinal deformities in NF1 patients.

**Supplemental table S1:** Prevalence of spinal abnormalities in NF-1 patients and type of mutation of the NF1 gene.

**Supplemental table S2:** Prevalence of clinical symptoms in NF-1 patients and type of mutation of the NF1 gene.

**Supplemental Table S3:** Influence of spinal abnormalities on clinical symptoms.
