# Supplementary figures and images for: Phenotyping spinal abnormalities in patients with Neurofibromatosis type 1 using whole-body MRI
Source: Sci Rep. 2021 Aug 19;11:16889. doi: 10.1038/s41598-021-96310-x (PMC8376946; doi:10.1038/s41598-021-96310-x)

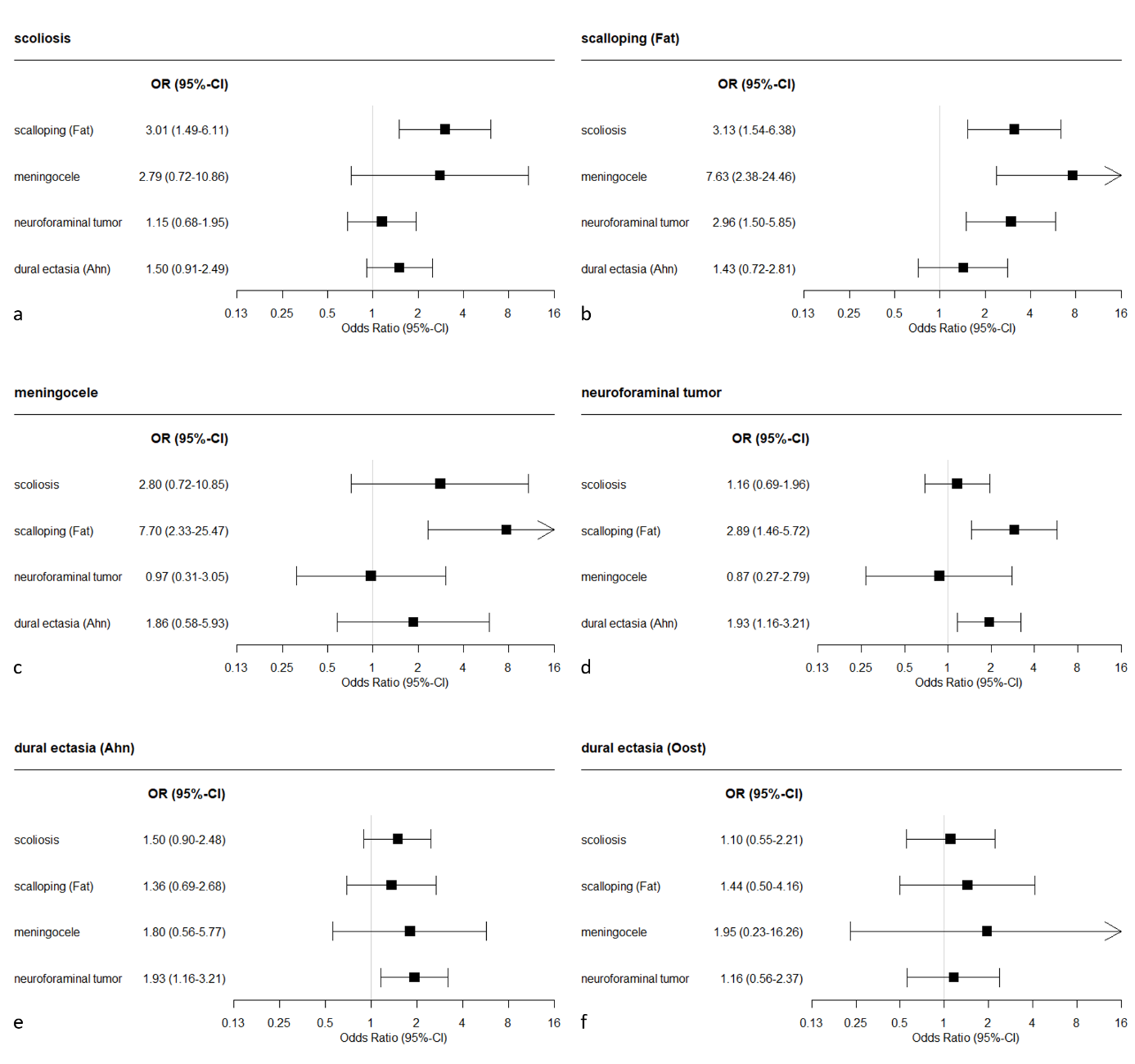

Supplement: Supplementary file 2 — Supplementary Figure S1. [file 41598_2021_96310_MOESM2_ESM.tif]
