# Supplementary material for: Phenotyping spinal abnormalities in patients with Neurofibromatosis type 1 using whole-body MRI
Source: Sci Rep. 2021 Aug 19;11:16889. doi: 10.1038/s41598-021-96310-x (PMC8376946; doi:10.1038/s41598-021-96310-x)
Supplement: Supplementary file 3 — Supplementary Table S1. [file 41598_2021_96310_MOESM3_ESM.docx]

**Supplemental table 1:** Prevalence of spinal abnormalities in NF-1 patients and type of mutation of the *NF1* gene.

| **items** |  | **group 1**  **(type 1 del)** | **group 2**  **(splice)** | **group 3**  **(missense)** | **group 4**  **(ns/fs)** | **group 5**  **(no proof)** | **group 6**  **(no analysis)** | **age** | **sex** |
| --- | --- | --- | --- | --- | --- | --- | --- | --- | --- |
| **scoliosis** | **prevalence** | 50% (9/18) | 50% (8/16) | 50% (17/34) | 46.4% (45/97) | 41.4% (29/70) | 52.5% (21/40) | - | - |
|  | **odds ratio**  **(95% CI)** | 1.43  (0.5 – 4.1) | 1.32  (0.43 – 4.01) | 1.34  (0.58 – 3.11) | 1.18  (0.63 – 2.22) | - | - | 1.02  (1.00 – 1.04) | 1.09  (0.64 – 1.86) |
|  | **p** | 0.51 | 0.63 | 0.49 | 0.6 |  |  | 0.023 | 0.75 |
| **meningocele** | **prevalence** | 5.6% (1/18) | 6.3% (1/16) | 5.9% (2/34) | 6.2% (6/97) | 4.3% (3/70) | 2.5% (1/40) | - | - |
|  | **odds ratio**  **(95% CI)** | 2.8  (0.43 – 18.34) | 1.78  (0.17 – 18.86) | 1.27  (0.2 – 8.12) | 1.5  (0.36 – 6.24) | - | - | 0.99  (0.96 – 1.03) | 0.53  (0.17 – 1.69) |
|  | **p** | 0.28 | 0.63 | 0.8 | 0.58 |  |  | 0.72 | 0.28 |
| **dural ectasia_(Ahn)_** | **prevalence** | 61.1% (11/18) | 68.8% (11/16) | 32.4% (11/34) | 44.3% (43/97) | 41.4% (29/70) | 45% (18/40) | - | - |
|  | **odds ratio**  **(95% CI)** | 2.56  (0.81 – 8.07) | 2.78  (0.8 – 9.67) | 0.6  (0.24 – 1.51) | 1.03  (0.53 – 2.03) | - | - | 1.05  (1.03 – 1.07) | 1.81  (1.01 – 3.24) |
|  | **p** | 0.11 | 0.11 | 0.27 | 0.92 |  |  | <0.001 | 0.045 |
| **dural ectasia_(Oost)_** | **prevalence** | 72.2% (13/18) | 87.5% (14/16) | 94.1% (32/34) | 83.5% (81/97) | 90% (63/70) | 80% (32/40) | - | - |
|  | **odds ratio**  **(95% CI)** | 0.28  (0.07 – 1.05) | 1.00  (0.18 – 5.53) | 1.68  (0.32 – 8.73) | 0.58  (0.22 – 1.52) | - | - | 0.98  (0.95 – 1.00) | 0.49  (0.22 – 1.1) |
|  | **p** | 0.06 | 0.99 | 0.54 | 0.27 |  |  | 0.041 | 0.084 |
| **neuroforaminal tumor** | **prevalence** | 38.9% (7/18) | 43.8% (7/16) | 44.1% (15/34) | 40.2% (39/97) | 37.1% (26/70) | 40% (16/40) | - | - |
|  | **odds ratio**  **(95% CI)** | 1.08  (0.37 – 3.16) | 1.14  (0.37 – 3.48) | 1.48  (0.63 – 3.46) | 1.13  (0.6 – 2.14) | - | - | 1.0  (0.99 – 1.02) | 1.73  (1.01 – 2.99) |
|  | **p** | 0.89 | 0.82 | 0.37 | 0.71 |  |  | 0.6 | 0.047 |
| **spinal tumor** | **prevalence** | 27.8% (5/18) | 12.5% (2/16) | 11.8% (4/34) | 11.3% (11/97) | 15.7% (11/70) | 7.5% (3/40) | - | - |
|  | **odds ratio**  **(95% CI)** | 2.12  (0.61 – 7.37) | 0.59  (0.11 – 3.08) | 0.75  (0.21 – 2.62) | 0.64  (0.26 – 1.6) | - | - | 1.03  (1.00 – 1.05) | 1.99  (0.9 – 4.41) |
|  | **p** | 0.24 | 0.53 | 0.65 | 0.34 |  |  | 0.026 | 0.088 |
| **scalloping**_(Fat)_ | **prevalence** | 16.7% (3/18) | 18.8% (3/16) | 23.5% (8/34) | 18.6% (18/97) | 17.1% (12/70) | 17.5% (7/40) | - | - |
|  | **odds ratio**  **(95% CI)** | 1.0  (0.2 – 3.9) | 1.1  (0.3 – 4.7) | 1.4  (0.5 – 3.9) | 1.1  (0.5 – 2.4) | - | - | 1.0  (0.99 – 1.03) | 0.9  (0.5 – 1.8) |
|  | **p** | 0.96 | 0.86 | 0.49 | 0.84 |  |  | 0.57 | 0.73 |
| **vertebral fracture** | **prevalence** | 5.6% (1/18) | 6.3% (1/16) | 8.8% (3/34) | 6.2% (6/97) | 7.1% (5/70) | 7.5% (3/40) | - | - |
|  | **odds ratio**  **(95% CI)** | 0.71  (0.07 – 6.95) | 0.53  (0.05 – 5.32) | 1.45  (0.31 – 6.86) | 0.77  (0.22 – 2.73) | - | - | 1.05  (1.01 – 1.08) | 3.54  (1.09 – 11.46) |
|  | **p** | 0.77 | 0.59 | 0.64 | 0.69 |  |  | 0.005 | 0.035 |
| **syringomyelia** | **prevalence** | 5.6% (1/18) | 0% (0/16) | 2.9% (1/34) | 4.1% (4/97) | 4.3% (3/70) | 2.5% (1/40) | - | - |
|  | **odds ratio**  **(95% CI)** | 1.63  (0.23 – 11.71) | 0.68  (0.03 – 13.9) | 0.77  (0.11 – 5.37) | 0.94  (0.23 – 3.79) | - | - | 0.99  (0.96 – 1.03) | 0.55  (0.16 – 1.88) |
|  | **p** | 0.63 | 0.8 | 0.79 | 0.93 |  |  | 0.73 | 0.34 |
| **herniation of intervertebral disc** | **prevalence** | 44.4% (8/18) | 43.8% (7/16) | 29.4% (10/34) | 26.8% (26/97) | 25.7% (18/70) | 17.5% (7/40) | - | - |
|  | **odds ratio**  **(95%)** | 2.42  (0.8 – 7.32) | 1.86  (0.58 – 5.97) | 1.25  (0.49 – 3.21) | 1.0  (0.49 – 2.06) | - | - | 1.03  (1.01 – 1.05) | 1.85  (1.01 – 3.39) |
|  | **p** | 0.12 | 0.3 | 0.64 | 0.99 |  |  | 0.004 | 0.045 |

Values in parentheses are numbers. Spinal abnormalities represent the dependent variable, respectively.

Ns/fs: nonsense/frameshift mutation
