# Supplementary material for: Phenotyping spinal abnormalities in patients with Neurofibromatosis type 1 using whole-body MRI
Source: Sci Rep. 2021 Aug 19;11:16889. doi: 10.1038/s41598-021-96310-x (PMC8376946; doi:10.1038/s41598-021-96310-x)
Supplement: Supplementary file 4 — Supplementary Table S2. [file 41598_2021_96310_MOESM4_ESM.docx]

**Supplemental table 2:** Prevalence of clinical symptoms in NF-1 patients and type of mutation of the *NF1* gene.

| **items** |  | **group 1**  **(type 1 del)** | **group 2**  **(splice)** | **group 3**  **(missense)** | **group 4**  **(nonsense/**  **frameshift)** | **group 5**  **(no proof)** | **group 6**  **(no analysis)** | **age** | **sex** |
| --- | --- | --- | --- | --- | --- | --- | --- | --- | --- |
| **pain** | **prevalence** | 22.2% (4/18) | 31.3% (5/16) | 14.7% (5/34) | 22.7% (22/97) | 25.1% (18/70) | 25% (10/40) | - | - |
|  | **odds ratio**  **(95% CI)** | 0.82  (0.23 – 2.87) | 1.21  (0.36 – 4.1) | 0.45  (0.15 – 1.38) | 0.8  (0.38 – 1.66) | - | - | 1.01  (1.01 – 1.05) | 1.07  (0.57 – 2.04) |
|  | **p** | 0.75 | 0.76 | 0.16 | 0.55 |  |  | 0.006 | 0.83 |
| **loss of motor function** | **prevalence** | 5.6% (1/18) | 6.3% (1/16) | 11.8% (4/34) | 9.3% (9/97) | 11.4% (8/70) | 10% (4/40) | - | - |
|  | **odds ratio**  **(95% CI)** | 0.44  (0.05 – 3.87) | 0.38  (0.04 – 3.37) | 1.25  (0.33 – 4.66) | 0.76  (0.27 – 2.12) | - | - | 1.01  (0.98 – 1.04) | 3.06  (1.17 – 8.02) |
|  | **p** | 0.46 | 0.38 | 0.74 | 0.6 |  |  | 0.37 | 0.023 |
| **loss of sensitivity** | **prevalence** | 0% (0/18) | 0% (0/16) | 2.9% (1/34) | 4.1% (4/97) | 8.6% (6/70) | 10% (4/40) | - | - |
|  | **odds ratio**  **(95% CI)** | 0.27  (0.01 – 4.9) | 0.32  (0.02 – 6.03) | 0.44  (0.07 – 2.73) | 0.49  (0.14 – 1.65) | - | - | 0.99  (0.96 – 1.03) | 0.86  (0.28 – 2.59) |
|  | **p** | 0.38 | 0.45 | 0.38 | 0.25 |  |  | 0.64 | 0.78 |

Values in parentheses are numbers. Clinical symptoms represent the dependent variable, respectively.
