# Supplementary material for: Phenotyping spinal abnormalities in patients with Neurofibromatosis type 1 using whole-body MRI
Source: Sci Rep. 2021 Aug 19;11:16889. doi: 10.1038/s41598-021-96310-x (PMC8376946; doi:10.1038/s41598-021-96310-x)
Supplement: Supplementary file 5 — Supplementary Table S3. [file 41598_2021_96310_MOESM5_ESM.docx]

**Supplemental Table S3:** Influence of spinal abnormalities on clinical symptoms.

| **items** |  | **scoliosis** | **meningocele** | **dural ectasia_(Ahn)_** | **neuroforaminal**  **tumor** | **spinal tumor** | **scalloping** | **syringomyelia** | **herniation of intervertebral disc** |
| --- | --- | --- | --- | --- | --- | --- | --- | --- | --- |
| **pain** | **prevalence** | 64.1% (41/64) | 23.4% (15/64) | 54.7% (35/64) | 73.4% (47/64) | 34.4% (22/64) | 39.1% (25/64) | 7.8% (5/64) | 29.7% (19/64) |
|  | **odds ratio** | 1.88  (0.95 – 3.73) | 2.41  (0.7 – 8.25) | 0.94  (0.48 – 1.86) | 3.8  (1.81 – 7.94) | 4.74  (1.92 – 11.72) | 2.66  (1.22 – 5.82) | 1.79  (0.38 – 8.35) | 0.72  (0.34 – 1.56) |
|  | **p** | 0.07 | 0.16 | 0.86 | <0.001 | 0.001 | 0.014 | 0.46 | 0.41 |
| **loss of motor function** | **prevalence** | 59.3% (16/27) | 14.8% (4/27) | 59.3% (16/27) | 92.6% (25/27) | 63% (17/27) | 55.6% (15/27) | 14.8% (4/27) | 29.6% (8/27) |
|  | **odds ratio** | 0.94  (0.31 – 2.85) | 3.44  (0.64 – 18.45) | 0.63  (0.2 – 1.95) | 6.76  (1.3 – 34.84) | 26.94  (6.54 – 110.9) | 7.91  (2.18 – 28.65) | 5.0  (0.72 – 34.83) | 0.47  (0.14 – 1.63) |
|  | **p** | 0.91 | 0.15 | 0.42 | 0.022 | <0.001 | 0.002 | 0.1 | 0.24 |
| **loss of sensitivity** | **prevalence** | 33.3% (5/15) | 6.7% (1/15) | 60% (9/15) | 86.7% (13/15) | 46.7% (7/15) | 33.3% (5/15) | 13.3% (2/15) | 33.3% (5/15) |
|  | **odds ratio** | 0.34  (0.1 – 1.13) | 0.98  (0.09 – 10.93) | 1.17  (0.35 – 3.95) | 7.06  (1.33 – 37.52) | 3.56  (1.0 – 12.64) | 1.38  (0.36 – 5.32) | 3.16  (0.5 – 20.15) | 0.67  (0.19 – 2.39) |
|  | **p** | 0.08 | 0.99 | 0.8 | 0.022 | 0.05 | 0.64 | 0.22 | 0.54 |

Pain, loss of motor function and loss of sensitivity represent the dependent variable, respectively.
